# Training Empathetic Communication Skills in Medical Students With a Role-Prompted GPT-4o Chatbot: Quasi-Experimental Intervention Study

**DOI:** 10.2196/88092

**Published:** 2026-06-12

**Authors:** Yannik Müller, Hans Lemberg, Paul Gellert, Jan C Zoellick

**Affiliations:** 1 Institute of Medical Sociology and Rehabilitation Science Charité - Universitätsmedizin Berlin Berlin, Berlin Germany

**Keywords:** artificial intelligence, AI, generative pretrained transformer, ChatGPT, chatbot, medical education, empathy, feedback, didactic method, educational tool, simulated patient

## Abstract

**Background:**

There is growing concern that artificial intelligence (AI) may diminish the quality of human relationships. However, in a context of widespread social importance (empathetic conversations between doctors and patients), AI can actually improve human conversational skills, potentially enhancing professional relationships. Recent advances in AI allow for realistically role-prompted counterparts for practicing professional conversations, enabling relational learning without the need for human counterparts.

**Objective:**

This study aimed to show the effectiveness of AI chatbots for learning professional communicative skills in medical education. Specifically, we hypothesized that a single conversation with an AI chatbot improves communication skills in medical students across 4 different conversational competencies.

**Methods:**

We conducted a quasi-experimental intervention study involving 4 distinct role-prompted scenarios (ie, shared decision-making, motivational interviewing, sexually transmitted diseases, and breaking bad news)—each designed to elicit in-depth empathic conversational skills aligned with key learning objectives in medical curricula. Students rated their competence for the 4 scenarios before and after a conversation with GPT-4o (OpenAI) using default settings, without fine-tuning. We expected higher perceived communication competence (PCC) in their conversation topic after the interaction compared with before the interaction in a 2-sided paired *t* test. Participants received AI-generated feedback, which they rated regarding adequacy. Post hoc analyses addressed gender and case effects, feedback adequacy, and prevalues in PCC.

**Results:**

This study shows that a role-prompted GPT chatbot improves PCC in 162 medical students after a single conversation with mean of 13 (SD 4.8; 95% CI 12-14) prompt-response pairs. We found an increase in PCC with a mean difference of 0.94 (SD 1.64; 95% CI 0.69-1.20; Cohen *d*=0.58) from 5.89 (95% CI 5.55-6.23; scale 0-10) before the conversation to 6.83 (95% CI 6.55-7.12) after the conversation across 4 different patient role prompts. Furthermore, we found participants rating AI feedback of their conversation to be useful (mean 7.92, SD 1.61; 95% CI 7.67-8.17; scale 0-10), but feedback adequacy did not correspond to PCC increase (*r*=0.08; *P*=.32).

**Conclusions:**

Our results demonstrate how role-prompted GPT increases self-assessed communication competencies, introducing a novel tool for teaching relational learning. Our results present a starting point for using AI in education, particularly teaching communication in professional roles. On the basis of our findings in medical education, we anticipate further studies to investigate conversational training between lawyers and clients, marketers and customers, or managers and employees. Our research thus has implications for any field with a need for conversational training and relational learning.

## Introduction

### Background

Communicating effectively and empathetically in professional roles is a key competence that builds trust, fosters empathy, and enhances outcomes for the parties involved. In medicine particularly, good communication leads to better adherence [1] and better health outcomes for patients [2,3]. Thus, teaching communication competencies is a core learning objective in contemporary medical curricula [4,5]. Conventional didactic methods to teach communication combine experiential and cognitive aspects and include (videotaped) observations, roleplays, actors as standardized or simulated patients, or real patient interactions [6-8]. However, those methods are resource-intensive and time-consuming. Evidence for the effectiveness of digital didactic formats continues to be weak [9].

Generative artificial intelligence (AI) chatbots have the potential to fundamentally change conversational patterns and human interpersonal relationships. Chatbots, described as “stochastic parrots” [10], might induce psychosis in vulnerable populations [11,12]: they “hallucinate” (ie, spread false information as credible) [13], reduce social interactions in professional relationships [14], or make established assessments such as essays insufficient in educational settings [15]. However, promising aspects have also been discussed. AI chatbots potentially reduce conspiracy beliefs [16] and climate change skepticism [17], provide correct medical information with high accuracy [18-20], and increase efficiency in administrative processes, such as discharging patients [21], writing medical reports [22,23], or analyzing medical notes [24].

In this study, we focus on AI chatbots as a novel didactic tool for communication competence. Previous proof-of-concept and feasibility studies have demonstrated that GPT technologies can be used as sophisticated role-prompted simulations in medical education [25-34]. However, these studies focused on single, confined tasks such as history taking or mammography, and their feedback—if provided at all—consisted of simple yes or no checklists of included topics diverging from behavior of actors, patients, or lecturers in educational settings. However, feedback is a central part of learning [35], particularly when presented in an adequate, resource-oriented fashion [36].

Building on these initial studies, we developed a tool that not only includes several simulated patient personas across various communicative techniques but also a feedback loop for performance on 8 different learning objectives as a didactic feature. The role prompts corresponded to core learning objectives in our university’s medical curricula. We hypothesized that a single conversation with an AI chatbot improves communication skills in medical students in 4 different conversational situations: shared decision-making (SDM) for treatment plans [37], motivational interviewing (MI) [38], sexual anamnesis and education [39], and breaking bad news [40]. We further investigated whether these competencies were domain-specific (ie, training with an SDM case only enhances SDM skills) or generalized (ie, training with SDM also enhances skills regarding MI, sexual anamnesis, and breaking bad news).

The purpose of this paper was to show the effectiveness and potential of AI chatbots for learning professional communicative skills in higher education. We used the GPT-4o model from OpenAI for building our tool with medical students from our university at different stages of their study.

### Key Innovations

Our approach complements and expands existing didactic tools in multiple ways. First, the tool offers a resource-efficient training tool that can be used by many students simultaneously anytime anywhere overcoming restrictions of time and space [41]. Second, the tool can be expanded easily by integrating other personas and use cases by inserting other system prompts. Third, while we focus on German language, the tool could easily adapt to any languages and cultural contexts included in the underlying GPT model. Such variability and expandability make our approach a viable tool that integrates easily into teaching and learning.

## Methods

### Study Design and Setting

We conducted a quasi-experimental intervention study assigning medical students to 1 of 4 intervention groups based on their semester of study to interact with an AI chatbot. For this purpose, we programmed a website for data collection and chatbot interaction called PythonAnywhere using Flask (version 3.1.0) in the backend [42]. The code is available upon reasonable request. Medical education in Germany consists of a 6-year program based on learning objectives that traditionally involves a preclinical (year 1 and 2), a clinical (years 3-5), and a practical phase (year 6) with state-regulated examinations [5,43]. Our medical faculty implements a reformed curriculum that integrates preclinical and clinical training with an emphasis on promoting communicative competencies. We developed 4 cases corresponding to core learning objectives in the German national competence-based learning objective catalog [5] that applies to all medical faculties: SDM, MI, sexual anamnesis, and breaking bad news. We mapped core learning objectives to these cases in Multimedia Appendix 1.

### Procedure

After encountering study information and providing informed consent, participants were asked questions on gender, semester of study, and the preinteraction outcome measure perceived communication competence (PCC) for all 4 topics to measure domain-specific or generalized skill development. On the basis of the semester of study, participants entered 1 of 4 chat dialogues: group 1 (semester 2 and 3) conversed with the chatbot prompted as a 58-year-old woman with a recent type 2 diabetes mellitus diagnosis regarding therapy options, group 2 (semester 4 and 5) used MI with a 58-year-old woman with chronic obstructive pulmonary disease indicating lifestyle changes, group 3 (semesters 6-8) performed a sexual anamnesis with a 25-year-old man with symptoms after practicing unprotected sex, and group 4 (semester 9 and higher) delivered the diagnosis lung cancer to a 58-year-old man, a heavy smoker with productive coughing who underwent computer tomography. All 4 system prompts can be found in Multimedia Appendix 1 alongside our prompt engineering process. For conversations with the chatbot, we used the application programming interface (API) for the GPT-4o model (default values for all parameters) [44]. We opted for GPT-4o as it represented the most advanced model during development. GPT-4o outperformed Anthropic Claude and Mistral AI Mixtral (we compared 4o to Claude Sonnet 4.0 and Mistral Large 2.1) in our initial tests. Participants had to give at least 4 inputs to ensure a minimum level of content for each conversation. We limited the number of messages sent via the API per conversation to 40 to cap resource use and avoid hour-long interactions that would be insufficient simulations of clinical practice. For feedback, we also used the API for the GPT-4o model (temperature=0.7; default values for all other parameters) [44].

**Figure 1:**
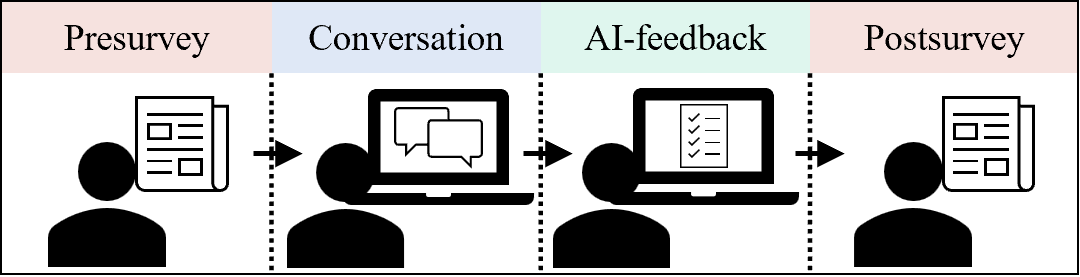
Study design. Participants displayed here as generic personas answered a presurvey, engaged in a conversation with an artificial intelligence (AI) chatbot, received AI feedback, and answered the postsurvey.

After interacting with the chatbot, participants immediately received feedback from GPT-4o using the case-specific feedback prompt found in Multimedia Appendix 1. The feedback had a standardized structure with a grade between 0% (very poor) and 100% (very good) followed by textual assessments across 8 criteria and a conclusion. Four general criteria applied to all cases (empathy, clear structure of the conversation, patient-centered approach, and open communication with open-ended questions), and 4 criteria were case-specific—always matched to the case participants experienced (eg, strengthening self-efficacy in case 1 or using MI techniques in case 2). The textual assessments often included quotes from the conversation and specific suggestions for improvement on the respective criterion. After receiving feedback, participants answered the 4 outcome questions again (postinteraction collection), and they evaluated the feedback using the perceived adequacy of feedback (PAF) scale [36]. We then asked participants to answer scales on technology acceptance [45,46] and the personality trait of technology commitment (TC) [47]. Finally, participants provided free comments on their experience during the study with the item “Do you have any additional feedback?” The study design is displayed in Figure 1.

### Outcomes and Assessments

We used the students’ PCC measured via preparation for a medical consultation as primary outcome obtained via self-reports of participants before and after the interaction with the chatbot (repeated measure) with the question “How well do you feel prepared for a medical consultation about...” rated for 4 topics on an 11-point Likert scale ranging between not prepared at all (0) and very well prepared (10). The 4 topics were (1) SDM for treatment plans, (2) MI, (3) sexually transmitted diseases, and (4) breaking bad news. We calculated the outcome as within-person difference postvalue minus prevalue.

The secondary outcome assessed the adequacy of AI feedback. We used the PAF subscale from the Feedback Perceptions Questionnaire [36] with 9 items rated on an 11-point Likert scale ranging between not at all useful (0) and extremely useful (10). After performing principal component and reliability analyses, we calculated a PAF mean score across all 9 items.

Our questionnaire also contained items on gender (male or female or diverse) and current semester of study (semesters 1-10 and practical year). Finally, we included instruments on technology acceptance. The simple scale for acceptance was a semantic differential with 9 items on a 5‑point Likert scale ranging between −2 and 2 [45]. Perceived usefulness (PU) and perceived ease of use (PEOU) from the technology acceptance model [46] each consisted of 4 items on a 5-point Likert scale ranging between completely disagree (score=1) and completely agree (score=5). Finally, TC encompassed 12 items on a 5-point Likert scale with 3 dimensions containing 4 items each ranging between completely disagree (score=1) and completely agree (score=5) [47]. After performing principal component and reliability analyses (acceptance: α=.87; PU: α=.93; PEOU: α=.94; TC [control beliefs]: α=.77; TC [competence]: α=.89; TC [acceptance]: α=.84), we calculated mean scores for each construct.

We obtained the length of the conversations by counting the number of messages and responses, and the number of characters written by the student and the chatbot, including spaces. We also obtained the AI-calculated grade between 0% (very poor) and 100% (very good) as part of the feedback.

### Sample Size

In our sample size calculation with nQuery+nTerim 4.0, we expected an average increase in PCC from a mean of 6.5 (before) to a mean of 7.0 (after) with equal SDs of 1.0 corresponding to a medium effect size (Cohen *d*=0.50) using established values for power (1-β=.80) and statistical significance (α=.05). Expecting a dropout of 10%, we formulated a recruitment target of 156 participants (ie, n=39 participants per case).

### Participants (Eligibility Criteria)

Our inclusion criteria were adult medical students enrolled at our university in study semesters 2 to 10. We excluded datasets that did not fulfill the inclusion criteria and those without responses to the primary outcome, multiple participations based on user and session IDs, or conversation topics outside of the intended medical case.

### Sampling Method and Recruitment Strategies

We used convenience sampling by inviting participants using internal student mailing lists and chatgroups enabling participation between June 17, 2025, and September 14, 2025.

### Statistical Analysis

We calculated descriptive statistics on primary and secondary outcomes. Regarding our primary hypothesis, we calculated a 2-sided paired *t* test between the prevalue and postvalue across all cases. We calculated additional 2-sided paired *t* tests between the prevalues and postvalues for each case individually with Bonferroni-Holm-adjusted significance levels. We additionally calculated a linear regression analysis with the difference between postvalue and prevalue as outcome and the prevalue in PCC as predictor to investigate the impact of prevalues on the learning increase. As secondary analyses, we calculated 2-sided independent *t* tests for gender differences in increased PCC, as well as correlations between the PCC scores, the AI feedback grade, the PAF score, and the acceptance measures. Finally, we identified the 5 AI feedbacks with the highest and lowest PAF scores for each case and contrasted their structural characteristics (ie, length and grade), as well as prevalues and pre-post changes for PCC in 2-sided independent *t* tests. We performed all data cleaning and analyses with MS Excel and SPSS (version 29.0; IBM Corp). All data can be found in Multimedia Appendix 2.

### Ethical Considerations

We preregistered our study design prior to data collection at AsPredicted (approval number 228674). The study was approved by the Charité Ethics Committee (EA1/038/24), and the study adhered to the Declaration of Helsinki. All participants gave informed consent.

## Results

### Sample

**Figure 2:**
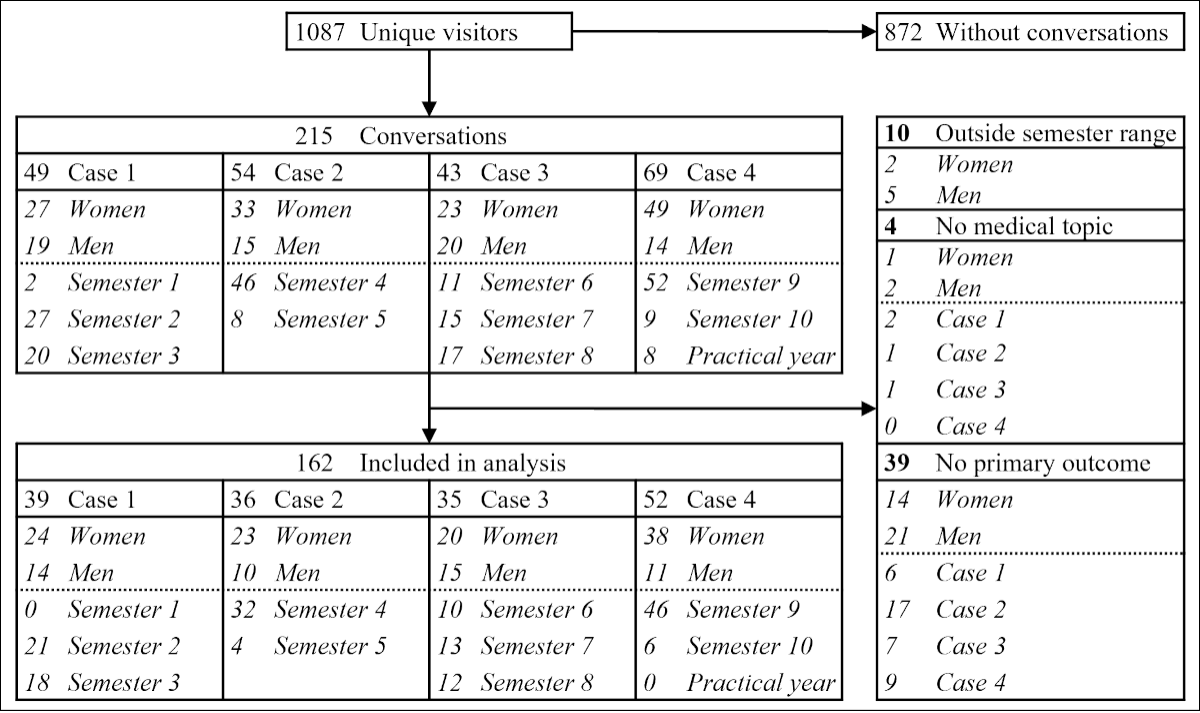
Study flowchart. For each case, we provided absolute numbers on gender and semester of participants. For each exclusion criteria, we provided absolute numbers for gender and assigned case. Semester 1 and practical year were outside the semester range. Missing data on gender account for differences between totals and sums.

A total of 1087 unique users visited the starting page of our website. Of those, 215 unique users engaged in conversations with the chatbot. We excluded 10 datasets from students outside the semester range and 4 datasets because they conversed about unintended topics. Finally, we excluded 39 datasets without postvalues for PCC. After exclusion, 162 unique users remained included in the analysis (Figure 2). Participants were mostly female (105/162, 65%) corresponding to the relative ratio of female students in our faculty (Table 1). Case-specific prevalues in PCC were the lowest in case 1 and the highest in case 4 mirroring study progression from beginning to end of the taught phase in medical studies. Participants reported high TC competence compared with the other dimensions of TC. Sensitivity analyses between 162 included datasets and 39 excluded datasets did not reveal differences regarding prevalues for PCC, number and length of messages, and grade in AI feedback (Multimedia Appendix 1). A total of 28 unique users engaged in 36 additional conversations with the chatbot, which we also excluded from our analysis.

**Table 1:** Sample characteristics (N=162).

Variables	Total	Case 1 (SDM^a^; n=39)	Case 2 (MI^b^, n=36)	Case 3 (STD^c^; n=35)	Case 4 (bad news; n=52)
Gender (woman), n (%)	105 (65)	24 (62)	23 (64)	20 (57)	38 (73)
Respective PCC^d^ prevalue, mean (SD)	5.89 (2.20)	4.64 (2.15)	5.78 (2.28)	5.91 (2.25)	6.88 (1.63)
TC^e^ control beliefs, mean (SD)	3.46 (0.77)	3.27 (0.83)	3.50 (0.81)	3.65 (0.84)	3.47 (0.62)
TC competence, mean (SD)	4.22 (0.85)	4.15 (1.05)	4.15 (0.85)	4.30 (0.79)	4.26 (0.74)
TC acceptance, mean (SD)	3.47 (0.91)	3.39 (1.09)	3.44 (0.88)	3.52 (0.76)	3.51 (0.90)

^a^SDM: shared decision-making.

^b^MI: motivational interviewing.

^c^STD: sexually transmitted disease.

^d^PCC: perceived communication competence.

^e^TC: technology commitment.

### Conversations

The conversations lasted for a median of 22 (IQR 16-30) minutes and contained on average 13 (SD 4.8) message pairs. Conversations in case 1 had few but long student prompts in contrast to the other 3 cases. Longer student prompts produced longer chatbot responses (*r*=0.59; *P*<.001). Descriptive statistics of the conversations and high-ranking and low-ranking exemplary conversations for each case are found in the Multimedia Appendix 1. We used around 5,400,000 input tokens and 370,000 output tokens for all conversations and feedback during data collection.

### Perceived Communication Competence

**Figure 3:**
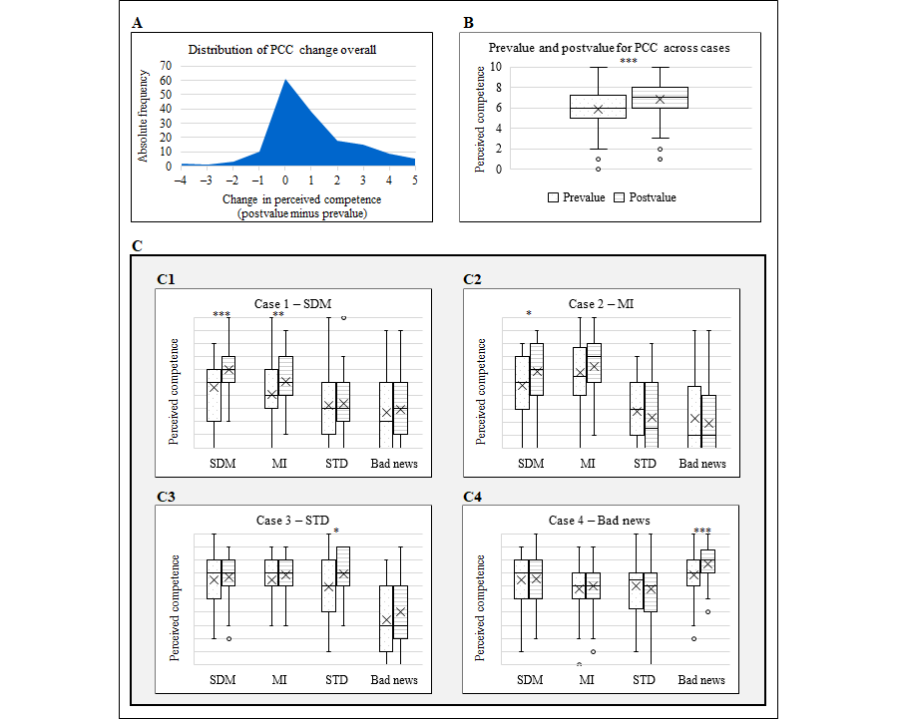
Graphical display of results for the primary outcome perceived communication competence (PCC): (A) distribution of change in PCC across all 4 cases, (B) boxplots for prevalue and postvalue PCC across all cases, and (C) case-specific boxplots for prevalue and postvalue PCC for all 4 interview situations. (C1) Prevalues and postvalues in PCC for all 4 interview situations of participants in case 1 (chatbot conversation about shared decision-making [SDM]), (C2) prevalues and postvalues in PCC for all 4 interview situations in case 2 (chatbot conversation about motivational interviewing [MI]), (C3) prevalue and postvalues in PCC for all 4 interview situations in case 3 (chatbot conversation about sexually transmitted diseases [STD]), and (C4) prevalues and postvalues in PCC for all 4 interview situations in case 4 (chatbot conversation about breaking bad news). Indicators for Bonferroni-Holm–corrected *P* values: *P≤.05, **P≤.01, ***P≤.001.

Participants reported higher PCC for the interview situation in their group’s topic after the conversation with the chatbot (mean 6.83, SD 1.84; 95% CI 6.55-7.12) than before the conversation (mean 5.89, SD 2.20; 95% CI 5.55-6.23; mean difference 0.94, SD 1.64; 95% CI 0.69-1.20; *t*_161_=7.33; *P*<.001; Cohen *d*=0.58). We observed increased PCC for the respective topic in cases 1 (mean difference 1.41, SD 1.77; *t*_38_=4.97; *P*<.001; Cohen *d*=0.80; n=39), 3 (mean difference 1.03, SD 1.89; *t*_34_=3.23; *P*=.001; Cohen *d*=0.55; n=35), and 4 (mean difference 0.85, SD 1.27; *t*_51_=4.79; *P*<.001; Cohen *d*=0.66; n=52), but not in case 2 (mean difference 0.50, SD 1.63; *t*_35_=1.84; *P=*.07; n=36). Figure 3 shows distributions and boxplots with prevalue and postvalues overall and for each of the 4 topics in each condition. We observed generalized PCC development in cases 1 and 2 with participants reporting increased perceived MI competence after conversing about SDM and vice versa. In contrast, participants in groups 3 and 4 experienced domain-specific skill development without generalization to other situations. Participants with lower prevalues reported higher increases in PCC in a linear regression analysis with post-pre difference as outcome and ln(prevalue) as predictor (B=−0.10, SE 0.008; β=−0.67; *t*_160_=−11.49; *P*<.001; *R*²=0.45).

### Feedback Adequacy

The feedback contained on average 3341 (SD 400) characters ranging in length between case 3 (mean 3139, SD 358) and case 4 (mean 3498, SD 473). Participants received an average grade of 83% (SD 5%) with no differences between cases. The feedback was generally perceived positively with an average PAF score of 7.92 (SD 1.61; scale 0-10). Participants rated feedback adequacy in case 1 the highest (mean 8.09, SD 1.66) compared with case 4 as the lowest (mean 7.67, SD 1.87). The PAF score did not correlate with the grade in the feedback (*r*=0.06; *P=*.44; n=156) or the length of the feedback (*r=*−0.01; *P=*.87; n=156).

Contrasting the highest- and lowest-ranked AI feedback of the 4 cases did not reveal differences regarding the length of the feedback (*t*_38_=0.33; *P=*.75) or the AI-generated grade (*t*_38_=−0.32; *P=*.75); participants did not differ regarding their prevalues (*t*_38_=0.61; *P=*.55) or their PCC gains (*t*_38_=−0.86; *P=*.40). Each of the 40 feedbacks followed the same structure, analyzing and highlighting improvements on the 8 categories.

Participants’ responses to the final open question of our survey regarding additional comments addressed generic AI phrases or the wish for specific and detailed examples for improvements. Some participants found the grade too harsh and not corresponding to the positive content of the text. However, participants providing these comments did not rank among the lowest PAF scores.

### Acceptance of Chatbot for Conversational Training

Participants reported high acceptance (mean 1.23, SD 0.55; scale −2 to 2), PU (mean 4.07, SD 0.89; scale 1-5), and PEOU (mean 4.56, SD 0.84; scale 1-5) after their interactions with the chatbot, without differences regarding cases, indicating similar usefulness and ease of use regardless of conversational situation. PCC increase correlated moderately with acceptance (*r*=0.29; *P*<.001) and PU (*r*=0.23; *P=*.004), but not with PEOU (*r*=0.03; *P=*.68). Other measures such as number of messages or characters as well as grade in the AI feedback did not correlate with acceptance (|*r*|≤0.11; *P*≥.15). Men and women did not differ regarding acceptance (*t*_151_=–1.05; *P*=.30), PU (*t*_150_=0.26; *P*=.80), or PEOU (*t*_150_=–0.56; *P*=.58). Table 2 shows subjective assessment of the chatbot in the 4 cases. Participants’ responses to the final open question of our survey regarding additional comments addressed the wish for spoken language instead of written text to include nonverbal cues, the unrealistic friendliness of the chatbot patient and its long responses, as well as technical barriers such as not being able to retract messages or sending messages too quickly by pressing enter.

**Table 2:** Descriptive statistics of acceptance, perceived usefulness (PU), and perceived ease of use (PEOU; n=160).

Variables	Total, mean (SD)	Case 1 (SDM^a^; n=38), mean (SD)	Case 2 (MI^b^; n=36), mean (SD)	Case 3 (STD^c^; n=35), mean (SD)	Case 4 (bad news; n=51), mean (SD)
Acceptance	1.23 (0.55)	1.28 (0.63)	1.07 (0.65)	1.34 (0.40)	1.23 (0.50)
PU	4.07 (0.89)	4.03 (0.98)	4.01 (0.92)	4.17 (0.95)	4.08 (0.76)
PEOU	4.56 (0.84)	4.46 (0.98)	4.42 (0.93)	4.69 (0.73)	4.65 (0.74)

^a^SDM: shared decision-making.

^b^MI: motivational interviewing.

^c^STD: sexually transmitted disease.

## Discussion

### Summary

Our study addresses the debate about harmful or beneficial effects of AI for interpersonal relations. Using highly emotive and professionally vital doctor-patient conversations, we demonstrate enhanced PCC across 4 scenarios requiring advanced skills and empathy. We thus provide evidence that an AI chatbot can be an effective didactic method to teach conversational skills. While we addressed medical students and their simulated patients, our tool targets high-level conversational skills also required in other professional relationships such as lawyers and clients, marketers and customers, or managers and employees. Specifically, we prompted GPT-4o as a simulated patient in 4 roles corresponding to core learning objectives in medical curricula with emotionally sensitive topics and a need for empathy (ie, SDM, MI, STDs, and breaking bad news). We combined this experiential component with structured feedback consisting of an overall grade for the student performance and text-based evaluations and improvements across 8 dimensions. The resulting tool is easily accessible, integrates seamlessly into the medical curricula, and complements existing didactic methods such as roleplays or actors simulating patients. Our aim was to increase students’ self-assessed competence in these scenarios after a single conversation with the AI chatbot compared to before. Analyzing data from 162 medical students, we demonstrated increased PCC scores overall and specifically in 3 of the 4 cases. Participants in the MI case did not report significantly better PCC regarding MI after exposure to the chatbot. We attribute these null findings to the comparatively high level of complexity of MI as conversational techniques, which makes achieving gains in PCC more challenging. Moreover, MI typically involves patients who express ambivalence, conflicting cognitions, and resistance, which are elements that a fundamentally supportive AI chatbot may struggle to represent adequately. The AI-generated feedback received positive evaluations by students regarding adequacy using the validated PAF scale. Contrary to studies addressing concerns [10-14], we demonstrate how AI chatbots can enhance communication skills fostering interpersonal relationships.

### Comparison With Prior Work

To the best of our knowledge, our tool is the first application of a GPT didactic tool for teaching professional communication skills in the broader sense. By that we mean an empathetic, patient-centered approach that uses open communication to develop specific, shared decisions for further medical procedures such as treatment or lifestyle interventions. Thus, we expand on use cases introduced in the study by Ning et al [41]. Accordingly, students in our study received detailed feedback that assessed the conversation across 8 dimensions providing improvements using specific examples from the interaction. Such a procedure enhances the learning experience of students because it contextualizes the assessment. This approach to communication and feedback sets our tool apart from previous feasibility or proof-of-concept studies that treated communication as a structured almost algorithmic task focusing solely on the information dimension [48]. For example, the most comparable study focused on history taking assessing the GPT conversations based on a list of topics included in the conversation (eg, main complaint, severity of symptoms, medication, or family history) [28]. Accordingly, their feedback consisted of a checklist of topics lacking a qualitative component assessing communication skills. Other studies did not include feedback at all but simply rated student performance in cases on chest and abdominal pain [27]. Finally, one other study used a similar approach to communication training as we did [32]. However, again, the focus of that study was on basic communication skills, such as history completeness and clinical reasoning, rather than social and emotional conversation competencies. We want to stress the importance of a holistic approach to communication as it encompasses those elements that build trust, create a shared understanding, and that are ultimately responsible for improving patient outcomes and experiences [1-3]. Indeed, the observed effects in PCC increase between case 1 (SDM) and case 2 (MI) demonstrate how empathy and goal-oriented guidance in medical conversations are generalized skills applicable to multiple situations expanding beyond a specific task.

### Strengths and Limitations

We built a chatbot as a didactic tool that integrates seamlessly in German medical curricula as it is based on core learning objectives relevant for all medical faculties. We matched cases to study progression addressing relevant learning objectives for each participant. The 4 cases cover several medical disciplines, and they combine general and domain-specific communication competencies. The gender distribution in our sample corresponds with our student population. We recruited a large sample size of students across the stages of medical studies. Thus, we are confident that our results can be generalized to other medical faculties in these regards. Cases might be matched to other semesters in medical faculties using the traditional curriculum. Selection bias potentially applies to technology affinity as more affine students likely participated in our study. However, we do not have data on technology affinity of nonparticipants or the student population in general.

Limitations of our study include the lack of a control group without exposure to our chatbot. We also used written text as mode of communication, which excludes nonverbal cues such as mimic, gestures, and prosody found to be informative in face-to-face interaction [49-53]. We opted for written text because this feature was cost-effective regarding OpenAI’s pricing model and it did not require participants to have audio input and output. We thus consider our findings a low bound estimate for training effects, and we expect studies with spoken language to increase PCC gains above the effect sizes we found in this study [32]. However, self-reports might also overstate short-term gains in PCC compared to external assessments. Conversations lasted 22 minutes on average exceeding time available for clinicians in practice. Furthermore, the grading system of AI feedback did not produce much variance. The AI feedback used only 17 grades out of 101 possibilities, and it provided the grade 85% in 92 of the 162 cases. We acknowledge that we recruited a homogeneous sample of students from the same university studying the same subject with the same curriculum, thus having a somewhat similar approach to patient interactions. However, we expected more variance in performance. In follow-up studies, we will thus continue to fine-tune the feedback prompt to create more variance in assessments. Finally, PCC as self-assessment provides relevant insights into student experiences and learning. Combining these self-assessments with external assessments in examinations with experienced lecturers expands the evaluation of AI chatbots.

### Future Research and Teaching

Our study with its preregistered effects and large sample size marks a shift from pilot studies to more advanced designs and approaches regarding chatbots in medical education. We propose future studies to follow more rigorous designs with randomization and control groups combining self-assessment with external assessment of learning outcomes, and varying difficulty and symptom severity of cases as well as AI models. For more realistic simulations of patient encounters, we suggest integrating spoken language and potentially avatars in a next step. Limiting interaction times to 5 to 10 minutes would realistically simulate clinical practice.

For teaching, our tool offers different deployment models: exclusive self-learning, preclass training and reflection, small-group debrief, or structured affiliation in a skills laboratory. In line with the human-in-the-loop paradigm, we suggest integrating AI conversations and feedback into the classes to discuss the interactions, their realism, and potentially irritating experiences resulting from AI hallucinations. We also propose a feedback loop for reporting irritating experiences to the technical chatbot deployment team for prompt modifications. Data protection and AI literacy is most relevant for students (eg, avoiding real names or personal information) as our proposed tool does not involve patient data.

### Conclusions

In conclusion, a single conversation with an AI chatbot role-prompted as a patient enhances students’ PCC across a variety of cases that represent core learning objectives in medical curricula. Our tool presents a novel didactic method that is versatile in its use and can thus be easily integrated into existing curricula. Potential deployment models for practical use include exclusive self-learning, preclass training and reflection, small-group debrief, or structured affiliation in a skills laboratory. Expanding the mode to spoken language integrated into a larger training regime offers potential for higher education in professional communication beyond medicine.

## Appendix

Multimedia Appendix 1 Prompts used and example conversations of all cases as well as sensitivity analyses.

Multimedia Appendix 2 Data used in our analyses.

## Abbreviations

AI: artificial intelligence
API: application programming interface
MI: motivational interviewing
PAF: perceived adequacy of feedback
PCC: perceived communication competence
PEOU: perceived ease of use
PU: perceived usefulness
SDM: shared decision-making
TC: technology commitment

## Data Availability

All data generated or analyzed during this study are included with this submission (and its multimedia appendices).
